# Extracellular vesicles: a potential new player in antibody-mediated rejection in lung allograft recipients

**DOI:** 10.3389/frtra.2023.1248987

**Published:** 2023-09-04

**Authors:** Sandhya Bansal, Ashwini Arjuna, Brian Franz, Alexa Guerrero-Alba, Jesse Canez, Timothy Fleming, Mohammad Rahman, Ramsey Hachem, T. Mohanakumar

**Affiliations:** ^1^ Norton Thoracic Institute, St. Joseph’s Hospital and Medical Center, Phoenix, AZ, United States; ^2^ HLA Laboratory, Vitalant, Phoenix, AZ, United States; ^3^ Department of Surgery, Washington University, St. Louis, MO, United States

**Keywords:** antibody mediated rejection (AMR), extracellular vesicle (EV), donor specific antibodies, lung transplanation, chronic lung allograft dysfunction (CLAD)

## Abstract

Identification of recipients with pre-existing antibodies and cross-matching of recipient sera with donor lymphocytes have reduced the incidence of antibody-mediated rejection (AMR) after human lung transplantation. However, AMR is still common and requires not only immediate intervention but also has long-term consequences including an increased risk of chronic lung allograft dysfunction (CLAD). The mechanisms resulting in AMR remain largely unknown due to the variation in clinical and histopathological features among lung transplant recipients; however, several reports have demonstrated a strong association between the development of antibodies against mismatched donor human leucocyte antigens [donor-specific antibodies (DSAs)] and AMR. In addition, the development of antibodies against lung self-antigens (K alpha1 tubulin and collagen V) also plays a vital role in AMR pathogenesis, either alone or in combination with DSAs. In the current article, we will review the existing literature regarding the association of DSAs with AMR, along with clinical diagnostic features and current treatment options for AMR. We will also discuss the role of extracellular vesicles (EVs) in the immune-related pathogenesis of AMR, which can lead to CLAD.

## 1. Introduction

Lung transplantation (LTx) is the primary treatment for patients with end-stage lung disease. Surgical advancements have improved outcomes, but the long-term function of the transplanted lungs remains disappointing, with a median survival after LTx of 6.2 years (1–4). Antibody-mediated rejection (AMR) of transplanted lungs remains an important problem, which is further complicated by a lack of consensus on the clinical characteristic as well as the immunological profile, histological features, and management strategies (5, 6).

Antibody-mediated rejection is a complex pathological, serological, and clinical process affecting graft function after transplantation. It has been better characterized in kidney and heart transplant recipients than in lung transplant recipients (LTxRs) (7). In LTxRs, specific B-cells and plasma cells producing antibodies directed against donor lung antigens can often be detected even before transplant, implicating these antibodies in the immunopathogenesis of AMR (8). Recent literature provides strong evidence of an important role for antibodies in AMR since antibodies to mismatched donor human leukocyte antigens, known as donor-specific antibodies (DSAs) and to lung self-antigens are often detected in patients with AMR. *De novo* production of DSAs can be detected within weeks to months after transplantation (6, 9, 10). Further, the presences of DSAs are associated with a poor prognosis and possibly accelerated graft failure, particularly within the first post-transplant year (11–13).

Investigations in the last few decades in solid organ transplants have demonstrated that antibodies, with or without a cellular response, could lead to ligation of major histocompatibility complex molecules, resulting in complement-dependent cell lysis with or without C4d deposition, which can damage the allograft (14–17). Other risk factors for AMR include gender; female recipients have higher risk of AMR post-transplant in cardiac patients (18–20); higher levels of pre-transplant panel reactive antibodies (PRAs) to HLA (21, 22); development of *de novo* DSAs resulting in positive donor-specific crossmatch (23); and re-transplantation (24). Per the International Society of Heart and Lung Transplantation (ISHLT) consensus, patients with AMR can be symptomatic (hypoxemia, decrease in FEV1, dyspnea, and pulmonary infiltrates) or asymptomatic (5, 25, 26). AMR can be clinical or subclinical with normal allograft function (25, 26), which can be further sub-categorized into definite, probable, and possible. These categories are based on the degree of certainty related to (a) pathologic, (b) serologic, (c) clinical, and (d) immunologic presentations (26).

The diagnosis of AMR in LTxRs is challenging as there is a lack of specific diagnostic criterion as well as tremendous variability in DSAs and allograft damage from patient to patient. There is a definite need to develop new diagnostic tools and techniques to diagnose and describe the clinical presentation of AMR, and ISHLT is currently attempting to come to a consensus on defining AMR (25, 27).

Chronic lung allograft dysfunction (CLAD) is the main barrier to good long-term outcomes the first year after lung transplantation (28, 29). Antibody-mediated rejection after lung transplantation is a progressive process that has been identified as a significant cause of morbidity that can lead to CLAD, eventually resulting in graft failure (5). In the current article, we will discuss recent updates on the understanding of AMR and our ongoing research on extracellular vesicles and their contents.

## 2. Pathogenies of AMR

Studies in kidney transplant recipients have helped to define the mechanisms of AMR (30, 31). AMR can be (a) hyperacute (occurring within minutes after the vascular anastomosis), (b) acute (occurring days to weeks after transplantation), (c) late acute (occurring within 3 months after transplantation), and (d) chronic (occurring months to years after transplantation) (5, 32).

Recent research has demonstrated that B cells and plasma cells produce DSAs that interact with the endothelium, leading to the activation of signaling pathways (31, 33). Antibody binding leads to the recruitment of immune cells leading to graft dysfunction, which can be either complement dependent or independent (34–36). The antibodies interacting with endothelium and activating different signaling pathways can be specific to HLA or non-HLA molecules (35, 36).

Non-HLA antibodies are further classified as alloantibodies and autoantibodies (37, 38). Unlike antibodies specific to HLA, non-HLA antibodies use alternative pathways to bind to endothelial cells causing injuries that do not involve binding to integrins (37). Fernandez et al. have demonstrated that one-third of lung recipients have pre-existing antibodies against non-HLA self-antigens, which can lead to hyperacute rejection (39). Subramanian et al. confirmed this viewpoint using a murine lung transplant model of rejection by administering antibodies specific to the lung self-antigen K alpha 1 tubulin (40).

## 3. Diagnosis of AMR and associated challenges

Allograft failure within 12 months of LTx due to AMR is one of the leading causes of early death in LTxRs (41). Combinations of multiple AMR diagnostic criterions are used at different centers. Several different invasive (biopsies, molecular microscopy) and noninvasive approaches (specific antibodies, donor-derived cell-free DNA, chemokine analysis, etc.) are used to diagnose AMR. As per ISHLT consensus on pulmonary AMR (25), a diagnosis of allograft dysfunction requires histologic evidence, complement component 4d (C4d) deposition, circulating DSAs, and the reasonable exclusion of other causes (11, 17). Although histology remains a popular diagnostic approach, new noninvasive methods involving blood (DSAs, donor-derived cell-free DNA, mRNA assays) or urine (chemokines, urinary mRNA, and urinary proteomics) are also becoming popular (5, 27, 42). We have summarized AMR diagnosis with invasive and non-invasive methods and treatments in Table 1.

**Table 1:** Types of antibody-mediated rejection, diagnosis, and treatment options.

Types	Diagnosis and clinical presentation	Treatment options
Acute antibody-mediated rejection/chronic antibody- mediated rejection	Invasive conventional methods: (a) Histologic evidence: Neutrophilic margination (43) Neutrophilic capillaritis Pulmonary capillaritis (44, 45) Septal capillary injury syndrome (46) Septal capillary necrosis (47). (b) C4d staining (47–49). (c) Detection of DSAs (HLA or non-HLA) in the serum Non-invasive newer methods (blood/urine): (a) donor-derived cell-free DNA measurements (blood) (b) Cytokine/chemokine measurements (blood/urine) (c) mRNA analysis (blood/urine) (d) Proteomics analysis (blood/urine)	Plasmapheresis—antibody removal from highly sensitized patients High-dose intravenous immunoglobulin Intravenous immunoglobulin and rituximab Plasmapheresis, intravenous immunoglobulin, and rituximab Complement inhibition Proteasome inhibitor

### 3.1. Histologic evidence

Lung graft dysfunction is associated with certain histological types. De Nicola et al. analyzed 41 biopsies from LTxRs with or without circulating DSAs. The authors concluded that pathological findings of grade 2+ neutrophilic infiltration is the most closely related to DSAs with graft dysfunction (50). Per ISHLT consensus (25, 27), the histopathologic features of AMR, which can progress to Chronic Lung Allograft Dysfunction (CLAD) include: neutrophilic margination (43), neutrophilic capillaritis, organizing pneumonia, pulmonary capillaritis (44, 45), septal capillary injury syndrome (46), and septal capillary necrosis (47). Immunohistochemistry for C4d, either by immunofluorescence (IF) or immunoperoxidase (IP) assays, may also provide supportive evidence of AMR (47–49).

### 3.2. C4d deposition

C4d is a degradation product of the complement pathway that binds to endothelium and is one of the markers of endothelial injury mediated by complement deposition (30). C4d deposition in recipients with AMR has been inconsistent and its role in the diagnosis has been controversial. The sensitivity of C4d deposition is always a question due to the emerging evidence of pulmonary AMR in the absence of C4d deposition (6, 51, 52). C4d staining has been difficult to interpret in lung biopsies because of poor reproducibility, high background staining, and poor specificity for AMR. Reports from the AMR studies in kidney and heart transplant recipients led to the recognition of a unique AMR pathogenesis, which is mediated primarily by natural killer cell interactions with DSAs, independent of complement activation and bound to endothelial cells (6, 53, 54). Studies from other organ transplant recipients will provide impactful insights in determining the incidence and clinical presentation of C4d-negative probable AMR and C4d-positive definite AMR after human lung transplantation (54, 55). Mauiyyedi et al. proposed that a correlation between C4d deposition and DSAs could be a diagnostic marker for AMR (56).

### 3.3. Donor-specific antibodies

Donor-specific antibodies have been strongly associated with acute allograft rejection after solid organ transplant, including kidney, heart, and lung (57–59). DSAs were first identified in 1960 in kidney transplant recipients undergoing AMR, and it was postulated that they may be associated with graft rejection/failure. Further evidence by other groups also supported this concept (59, 60). All nucleated cells within transplanted lungs can express HLA class I antigens (HLA-A, HLA-B, or HLA-C). In addition, antigen-presenting cells (APCs) within the lungs may also express HLA class II antigens (HLA-DQ, HLA-DR, HLA-DP) (61). In addition, the expression of HLA class II molecules can be induced on pulmonary endothelial cells in response to pro-inflammatory cytokines (62, 63).

DSAs have been associated with not only AMR but also the development of CLAD in lung transplant recipients, manifested as bronchiolitis obliterans syndrome (BOS) or, more frequently, restrictive allograft syndrome (RAS) (28, 29, 64).

Although there are number of traditional options to diagnose AMR (histologic evidence, C4d deposition, and DSAs), there are problems with the current identification methods: (a) positive C4d stains without detectable DSAs; (b) positive C4d stains without graft abnormalities; (c) variable levels of DSAs with varying strength; and (d) antibodies to non-HLAs with or without detectable DSAs, etc. Therefore, it is important to devlop modern diagnostic techniques to determine AMR. One emerging possibility, in addition to C4d staining, is non-invasive molecular diagnostics (27, 42).

## 4. Management and treatment of AMR

Despite the identification of AMR in lung, kidney, and heart transplant recipients, the literature describing the management of AMR after lung transplantation is very limited. Treatment strategies for AMR in LTxRs are based on experience with other solid organ transplants which varies by center. There are multiple treatment options that can be considered to treat AMR. The overall aim of AMR treatment is to deplete the circulating antibodies, plasma cells, and B-cells to decrease antibody-mediated graft injury. Combinations of therapies have also been demonstrated in different centers that include plasmapheresis, intravenous immune globulin (IVIG), plasma cell depleting agents, T- or B-cell specific agents, and targeting of the complement pathway.

### 4.1. Plasmapheresis and intravenous immune globulins

Plasmapheresis and intravenous immune globulins remove antibodies but also reduce cytokine levels. Multiple studies have shown better graft function with the combination of plasma exchange and intravenous immune globulins (IVIG) (65, 66). Plasmapheresis is used to remove or reduce the level of existing antibodies by replacing patient plasma with plasma from healthy individuals. IVIG therapy decreases HLA sensitivity, which in turn lowers the level of HLA antibodies by blocking their ability to attack the transplanted organ.

### 4.2. Anti-CD20 antibody

Rituximab, an anti-CD20 monoclonal antibody, has been used for AMR treatment. Rituximab binds to pre-B-cells and mature B-lymphocytes that express CD20 (67).

### 4.3. Complement inhibition

Eculizumab, a monoclonal antibody, is used to inhibit complement component C5 during the formation of the membrane-attack complex, which is the final common pathway of AMR (68).

Although there are multiple treatment options for AMR, the combination of treatments depends on the patient′s status, and center bias still holds dominance. To date, there is no uniformity or consensus treatment for AMR. Different centers are using different combinations of treatments depending on their results. There is a need for more clinical trials, and studies are needed to address the impact of gender, demographics, and populations in different locations (69).

## 5. Extracellular vesicles

**Figure 1:**
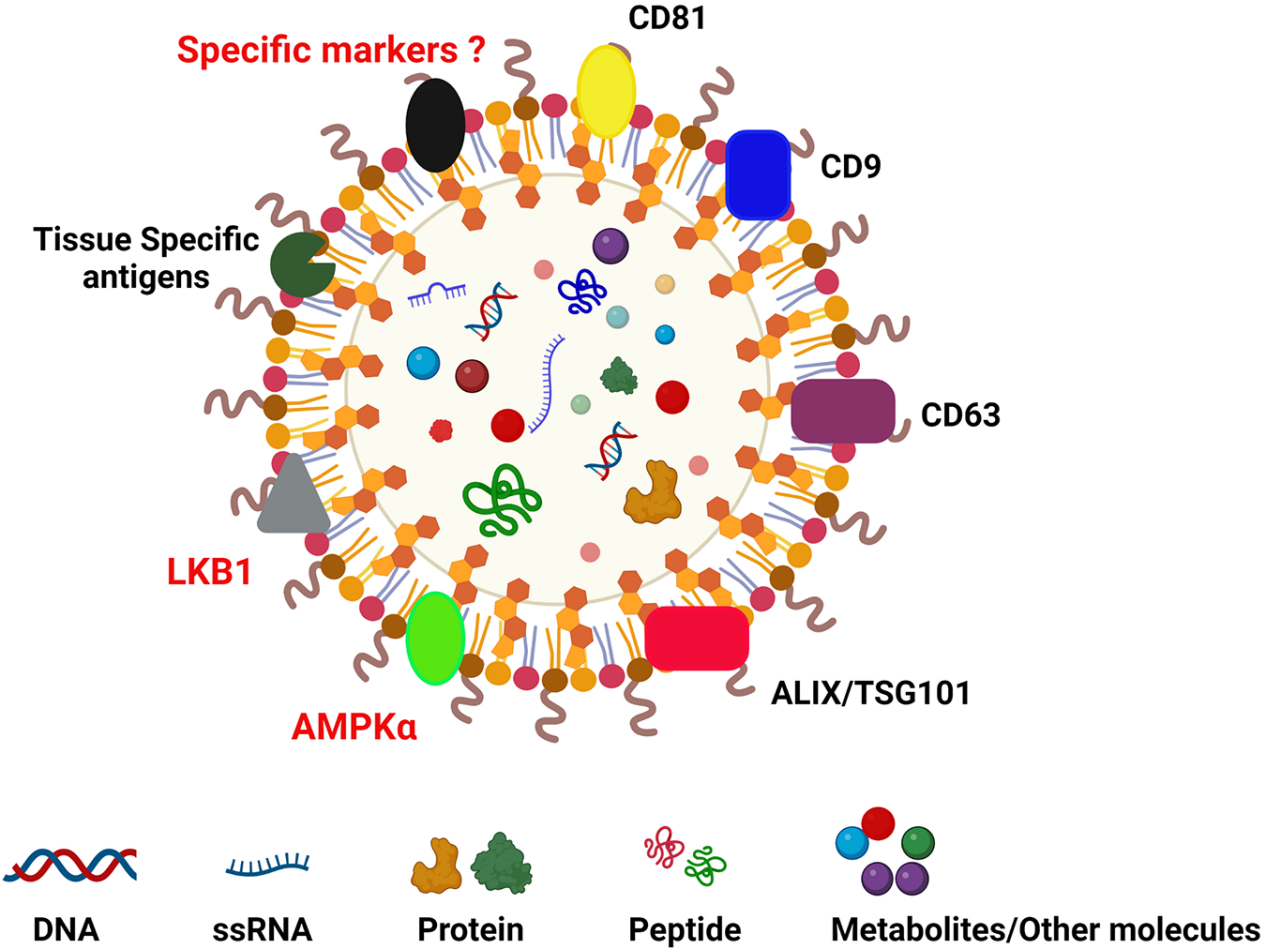
Diagrammatic reprentation of extracellular vesicle carrying CD9, CD63, CD81, TSG101, ALIX, tissue-specific markers (collagen V and K alpha1 tubulin in lung transplant; cardiac myosin and vimentin in cardiac transplant; fibronectin, collagen IV and perlecan in kidney transplant) (73). Images created using BioRENDER.

Extracellular vesicles (EVs) are a group of particles that are encapsulated by a lipid bilayer; they are released from different types of cells in the body and are present in body fluids (70). The origin of EVs can be ectosomal or endosomal. They are divided on the basis of size: (a) microvesicles (100–1,000 nm); (b) apoptotic bodies (1,000–5,000 nm); (c) exosomes/small EVs <200 nm (71, 72) (Figure 1). There are multiple terms associated with the nomenclature of EVs and exosomes as per Minimal Information for Studies of Extracellular Vesicles (MISEV) guidelines. Research to identify biomarkers specific to different EVs is ongoing, but currently there are no specific markers associated with EV subtypes. There are different kinds of classifications: (A) based on origin of EVs. EVs originated from endosomes are called “exosomes” and the EVs which are plasma membrane-derived are referred to as “ectosomes”: (B) Based on size, small EVs (<200 nm) and medium/large EVs (>200 nm); (C) on the basis of density low, medium and high; and (D) on the basis of composition CD9/CD63/CD81 etc. (74). Still newer guidelines are needed for the uniformity of the terms related to EVs. Witwer et al. have explained the different nomenclatures of EVs and what is influencing the choice of authors (75). We will be using the term EVs in the current manuscript. The composition of EVs depends on the cells releasing them. This can vary depending upon on the clinical status of the patient (acute and chronic rejection, respiratory viral infections, cancers, etc.) and many other clinical conditions (28, 76–78). EVs are encapsulated by a lipid bilayer and carry specific surface markers (e.g., CD9, CD81, Flotilin, HSP70, tetraspanins, Alix, CD63) along with major histocompatibility complex (MHC) molecules. EVs not only participate in intercellular communication but also have a role in pathological and physiological processes related to diseases.

### 5.1. EVs in solid organ transplantation

A large number of reports are available on the role of EVs in the activation and regulation of the immune system. It is highly possible that EVs carry intracellular and membrane proteins from the cells of origin, which makes them a potential candidate for biomarkers of various disease states. The ability of EVs to circulate in different bodily fluids (serum/plasma, saliva, urine, bronchial alveolar lavage, cerebrospinal fluid, etc.) makes them a less invasive biomarker compared to tissue biopsies. These biomarkers can be protein/peptide, DNA, RNA, or small metabolites (79–81).

Solid organ transplantation is the last option for patients with end-stage organ failure. Different groups have studied EVs in different solid organ transplant recipients to identify various biomarkers; this has been recently summarized by Garcia et al. (82). The details of EVs as biomarkers in lung transplant recipients are provided in Table 2. Our group is focused on lung transplant, thus we will discuss more about EVs in lung transplant recipients. Failure of normal lung function can be caused by several diseases including cystic fibrosis, idiopathic pulmonary fibrosis, chronic obstructive pulmonary disease, autoimmune disease, and respiratory infections (91). There are many advancements in the last decade in surgical strategies, but the outcomes are still poor (92, 93), and the median survival of LTxRs is limited to ∼5.8 years (41). Immune mechanisms are the driving force behind the development of rejection after transplantation. LTxRs who develop antibody-mediated rejection, have a higher chance of developing CLAD, which depends on the number and severity of early AMR episodes (83, 94).

**Table 2:** Extracellular vesicles as biomarkers in lung transplant recipients with different clinical conditions.

Condition			
CLAD/BOS/RAS	Respiratory viral infections	Antibody-mediated rejection	References
Lung self-antigens (collagen V and Kα1 tubulin), LKB1, AMPK1α, PIGR, HLA-DQ, HLA-DR miR-155, miR-142- 5p, TLR2, miR-182, miR-92a	Respiratory viral antigens [rhino, corona (HKU1, OC43, 229E, NL63, SARS-CoV-2 spike protein and nucleocapsid, respiratory syncytial virus], granzyme B, MST1	LKB1, AMPK1α (Not published) Shuttle RNA (esRNAs)	(28, 73, 76, 77, 83–90)

CLAD, chronic lung allograft rejection; BOS, bronchiolitis obliterans syndrome; RAS, restrictive allograft syndrome.

### 5.2. EVs in chronic lung allograft dysfunction

EVs can be potential biomarkers for LTxRs at risk for the development of CLAD (28, 83, 84). We have also presented evidence that EVs and their contents can differentiate between different phenotypes of CLAD (BOS and RAS) (28, 84). Our results have demonstrated significantly higher levels of lung self-antigens, transcription factors, 20S proteasome, polymeric immunoglobulin receptor (PIGR), and HLA antigens on the EVs from CLAD as compared to stable patients. We further delineated this in different phenotypes of CLAD (BOS/RAS) in a recent study, where we have demonstrated the significantly higher amounts of transcription factor NFkB, 20S Proteasome, PIGR, MHC Class I (W6/32) and II (HLA DQ, DR) in EVs from RAS phenotype of CLAD (28). In addition, mice immunized with EVs isolated from LTxRs with different CLAD phenotypes caused damage to mice lungs with varying severity and type of injury. Previous reports from our laboratory have shown that small EVs play a significant role in development of CLAD (28, 85) but the association of EVs with AMR has not been explored.

### 5.3. EVs in AMR

Extracellular vesicles are emerging as key biomarkers and mediators in several diseases by carrying specific markers involving both cell-cell interaction and regulation (73, 86, 95, 96). Franzin et al. have shown that EVs can play a pertinent role in tubular senescence and epithelial-to-mesenchymal transition in kidney transplant recipients with AMR (97). In this study authors have characterized the EVs from patients with AMR in Kidney transplants. They have published the data on the difference in presence of pro-inflammatory, pro-aging and profibrotic effects on tubular and endothelial cells in kidney transplant recipients with AMR and controls (No AMR). EVs from AMR patients carried significantly higher amounts of miRNAs which were associated with the renal inflammation, tubular senescence and renal fibrosis as compared to controls. They have also demonstrated that EVs from AMR patients induced Epithelial to mesenchymal transition by significantly decreasing the endothelial markers, such as CD31 and VE-Cadherin and increasing the fibroblast markers Vimentin and collagen I in the endothelial cells treated with EVs from AMR patients. Limited literature is available on EVs and their association with AMR after LTx.

**Figure 2:**
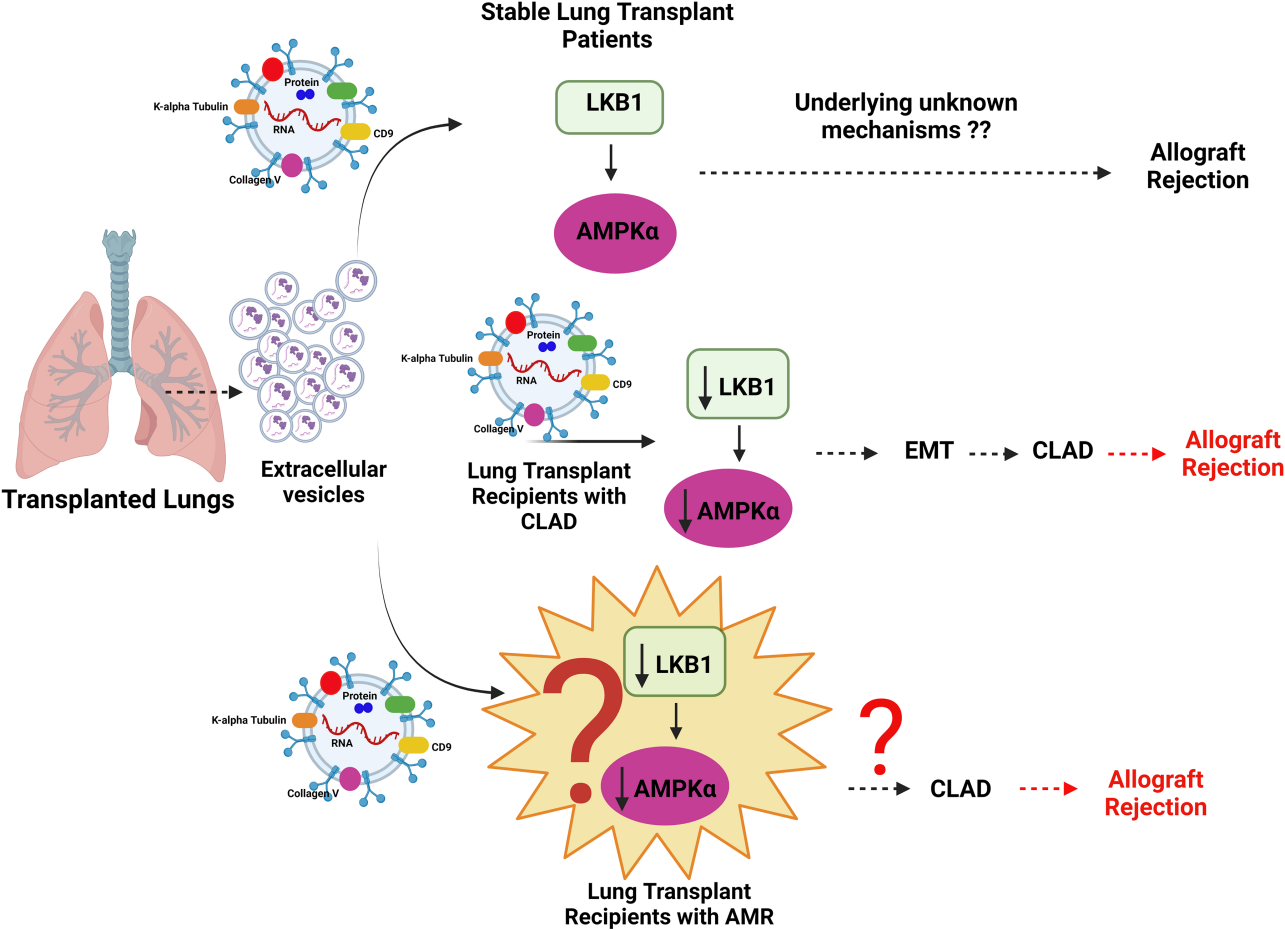
Diagrammatic representation of the mechanism of allograft rejection in lung transplant recipients (LTxRs) with antibody-mediated rejection (AMR). Extracellular vesicles from LTxRs with CLAD carry lower amounts of liver kinase B (LKB1) and AMPKα than those from stable LTxRs. Decreased levels of LKB1 downregulate AMPKα, which can potentially increase epithelial-to-mesenchymal transition followed by chronic lung allograft dysfunction and graft loss. Similarly EVs in AMR can also play a potential role involving LKB1 and AMPKα which can be further associated with development of CLAD followed by graft rejection, this hypothesis needs further investigation. Images created using BioRENDER.

Our preliminary data with LTxRs at the time of development of AMR have shown the presence of lower amounts of LKB1, and AMPK1α in EVs isolated from plasma as compared to stable controls (data not presented). Lower levels of LKB1 and AMPKα in EVs from LTxRs at the time of AMR is in agreement with the *in vitro* and *in vivo* studies conducted by Rahman et al. with samples from LTxRs with CLAD (87, 88). On the basis of our published data on CLAD and preliminary investigations with AMR in lung transplant patients (data not shown), there is a possibility that LKB1 and AMPK1α may play a key role in AMR and a biomarker for the development of CLAD. This hypothesis needs further investigation and validation. Rahman et al. have reported the role of the tumor suppressor gene LKB1 in the initiation of epithelial-to-mesenchymal transition resulting in CLAD after lung transplantation in humans and mice (87, 88). Based on our published data on LTxRs with CLAD and unpublished data on those with AMR, we propose LKB1 as a potential EV biomarker for the onset of AMR in LTxRs as shown in Figure 2. Future studies are needed to explore the novel mechanisms of interactions and the role of LKB1 in the pathogenesis of AMR.

## 6. Future directions

Due to a lack of experimental data regarding EVs and their underlying mechanisms in LTxRs with AMR, the scope of required EV biomarker research is huge. Comparing the levels of biomarkers in EVs from the plasma isolated from LTxRs before and after the onset of AMR may be predictive of clinical outcomes, i.e., CLAD. Biomarker analysis on EVs in LTx needs further investigation with a larger number of patient samples from multiple centers. In addition, the analysis of EVs needs to be expanded by studying the differences in biomarkers between DSA-positive and DSA-negative samples from LTxRs with AMR. A multi-parametric study considering pre-, current, and post-AMR along with DSA status and following up with CLAD development can help to elucidate the mechanisms of EVs.

## Data Availability

The original contributions presented in the study are included in the article/Supplementary Material, further inquiries can be directed to the corresponding author.
